# Evaluation of the Quick Environmental Exposure and Sensitivity Inventory in a Danish Population

**DOI:** 10.1155/2012/304314

**Published:** 2012-01-12

**Authors:** Sine Skovbjerg, Nikolaj Drimer Berg, Jesper Elberling, Karl Bang Christensen

**Affiliations:** ^1^The Danish Research Centre for Chemical Sensitivities, Department of Dermato-Allergology, Gentofte Hospital, University of Copenhagen, 2820 Gentofte, Denmark; ^2^Research Centre for Prevention and Health, Glostrup Hospital, University of Copenhagen, 2600 Glostrup, Denmark; ^3^Section of Biostatistics, Department of Public Health, University of Copenhagen, 1165 Copenhagen, Denmark

## Abstract

*Objectives*. To evaluate a Danish translation of the Quick Environmental Exposure and Sensitivity Inventory (QEESI). *Methods*. The study included two groups: one comprised a random sample of 2000 individuals drawn from the Danish Civil Registration System; the other comprised 315 patients with chemical intolerance. *Results*. The evaluation suggested good reliability for the four QEESI scales in terms of internal consistency and coefficients between test and retest scores. The discriminatory validity was the largest for the Chemical (inhalant) Intolerance and Life Impact Scales. Using combined cut-off scores for these two scales provided a sensitivity of 92.1 and a specificity of 91.8 and yielded a prevalence of 8.2% in the population group. *Conclusions*. The Danish translation of the QEESI showed overall good reliability and validity. We recommend the use of the combined Chemical (inhalant) Intolerance and Life Impact Scales in future studies.

## 1. Introduction

Chemical intolerance, also referred to as multiple chemical sensitivities (MCS), is a disorder characterized by reports of nonspecific symptoms from various organ systems attributed by the individual to exposure to common airborne chemicals [1]. In general the reported symptoms are attributed to previous chemical exposures and recur on subsequent exposures to the same or structurally unrelated chemicals at levels normally considered to be nontoxic [1]. 

Symptoms of chemical intolerance are prevalent with estimates ranging from 9 to 33% in population-based studies; however, such studies are few [2–7]. Physician-diagnosed MCS or reports of disabling consequences in the form of social and occupational disruptions attributed to exposure to common airborne chemicals range from 0.5 to 6.3% [2–4, 7]. The reported symptoms typically vary between individuals with women being more sensitive and reporting more symptoms than do men [2, 5–7]. A typical symptom pattern is thus difficult to establish. Nonspecific central nervous system (CNS) complaints are frequently reported, including fatigue, headache, and difficulty concentrating [2, 8, 9]. Other symptoms include pain and respiratory complaints [2, 5, 6]. An association between asthma and chemical intolerance has been reported in several studies [2, 10, 11]. In a population-based twin study on the heritability of perfume-related respiratory symptoms, Elberling and colleagues reported a heritability of 0.35 [12]. A mutual genetic correlation of 0.39 was reported for perfume-related respiratory symptoms and atopic dermatitis, suggesting some genetic pleiotropy for these two factors. No genetic pleiotropy was found between perfume-related respiratory symptoms, hand eczema, contact allergy, or asthma [12], suggesting that the association with asthma might be caused by mechanisms other than genetic susceptibility. Increasing evidence points to an association between MCS and symptoms of psychological distress, that is, depressive symptoms, somatisation, negative affect, and anxiety [13–18], which are likely to add to the level of overall functional disability.

The label “MCS” was initially proposed by Cullen based on clinical observations [19]. Although more case definitions have been proposed since the introduction of Cullen's criteria in 1987 [9, 20], none is currently widely accepted [20, 21]. The absence of widely accepted case criteria for establishing the presence and degree of chemical intolerance challenges epidemiological and clinical studies in this field. Several self-report questionnaires have been developed for research purposes [22–24]. The questionnaire that appears to have been most widely applied is the Quick Environmental Exposure and Sensitivity Inventory (QEESI) developed by Miller and Prihoda [25, 26]. QEESI is a reliable and valid self-administered questionnaire that was developed to gauge the multisystem symptoms and multiple intolerances often reported in chemical intolerance [25, 26]. QEESI consists of five scales measuring different domains related to chemical intolerance, that is, commonly reported symptoms, chemical (inhalant) intolerances, other intolerances (e.g., allergies, foods, alcohol), life impact attributed to chemical intolerances, and on-going exposures from routinely used products (Masking Index). Four of the QEESI scales consist of ten items where responses are rated on an eleven point scale ranging from “*not at all a problem”* (0) to *“disabling symptoms” *[10], resulting in a score range from 0 to 100. The fifth, the Masking Index, also consists of ten items, but the response format is dichotomous (0 or 1), resulting in a score range from 0 to 10. QEESI has been translated into a number of different languages, that is, Swedish [27], Japanese [28, 29], and Spanish [30], of which the Swedish and Japanese versions have also been evaluated in terms of validity and reliability. The Swedish study included a mildly (*n* = 67) and a moderately/severely chemically intolerant group (*n* = 126) and a control group (*n* = 90). The study concluded that the Swedish version of QEESI is reliable and valid for investigating chemical intolerance [27]. The Japanese study included a general population group (*n* = 498) and an outpatient group with self-reported MCS (*n* = 131) [28]. Based on principal components analyses, this study concluded that three of the QEESI subscales, that is, Symptom Severity, Chemical (inhalant) Intolerances, and Life Impact, were valid. To the best of our knowledge, no other study has established normative data based on a large population-based sample, and an evaluation of a Danish version of QEESI will not only strengthen future studies on chemical intolerance but will also enable international comparisons of data.

The objectives of the present study were (1) to evaluate a Danish translation of the QEESI in relation to validity and reliability, (2) to describe sensitivity and specificity, (3) to test whether asthma and high scores (based on Danish population norms) on SCL-92 subscales of depression and somatisation were associated with scores on QEESI, and (4) to establish normative data. 

## 2. Methods

### 2.1. Participants

Two groups were invited to participate in the study: (1) individuals from the general population and (2) individuals who had contacted the Danish Research Centre because of symptoms attributed to common airborne chemicals, and patients with physician-diagnosed chemical intolerance.

#### 2.1.1. General Population

A random sample of 18–69-year-old (*n* = 2000) from the general population was drawn from the Danish Civil Registration system in January 2010.

#### 2.1.2. Patients

The patient sample (*n* = 315) comprised individuals who had contacted the Danish Research Centre for Chemical Sensitivities between January 1, 2006 and January 1, 2010 (*n* = 183) because of reactions consistent with chemical intolerance, and individuals who had received a diagnosis of chemical intolerance either at the Copenhagen University Hospital, Rigshospitalet, or at Hamlet, Private Hospital, Denmark, between January 1, 1990 and January 1, 2009 (*n* = 132) by the same ear-nose-and throat specialist.

### 2.2. Measurements

#### 2.2.1. A Danish Translation of QEESI

The original version of QEESI [25, 26] was translated into Danish by a professional translation agency. The Danish translation was subsequently tailored to Danish usage and then translated back to the original language by a different professional translator. The back translation was then compared with the original version of QEESI to identify potential sense-altering discrepancies. Finally, the Danish translation was pilot tested among individuals with chemical intolerance for comprehension and ease of completion.

#### 2.2.2. Symptom Checklist 92

Symptom Checklist 92 (SCL-92) subscales for depression and somatisation were included. These subscales comprise 25 items where responses are rated on a 5-point Likert scale ranging from *not at all *to *very much*. The SCL-92 has been validated in a general Danish population and normative data have been established [31, 32].

#### 2.2.3. Asthma

Questions on asthma were adopted from the Stage 1 questionnaire of the European Community Respiratory Health Study (ECRHS) [33]. Asthma was defined according to criteria employed by the ECRHS as an affirmative answer to at least one of the following questions: (1) *Have you been woken by an attack of shortness of breath at any time in the last 12 months?* (2) *Have you had an attack of asthma in the last 12 months?* (3) *Are you currently taking any medicine (including inhalers, aerosols, or tablets) for asthma? *[34]. 

#### 2.2.4. Procedure

A questionnaire was sent to the participants on two occasions. The first test occasion included (1) the QEESI, (2) questions on socioeconomic position, categorized in accordance with the British Registrar General's Classification I–V [35], (3) the SCL-92 somatisation and depression subscales, and (4) the ECRHS asthma questions. Demographic data, for example, age and sex, were available. Two months after responding to the first questionnaire, a random sample of the respondents from the general population (*n* = 200) and 140 patients who had responded to the first questionnaire received a second questionnaire, which consisted of the QEESI only. The overall response rate to the first questionnaire was 64.5%. The response rates in the population sample were 65.3% (*n* = 1305/2000) on the first test occasion and 61.0% (*n* = 122/200) on the second test occasion. The response rates in the patient sample were 60.0% (*n* = 189/315) on the first test occasion and 80.0% (*n* = 112/140) on the second test occasion.

## 3. Statistical Analysis

### 3.1. Reliability and Validity

The internal consistency of the four QEESI scales (Chemical (inhalant) Intolerances, Symptom Severity, Other Intolerances, and Life Impact) was evaluated using Cronbach's alpha [36]. Coefficients were calculated for the patient sample and for age stratified samples of the population. Test-retest reliability was evaluated using Pearson correlations.

The discriminatory validity of the QEESI was evaluated using bivariate logistic regression, and multivariate analyses were also used for the Chemical (inhalant) Intolerance and Life Impact Scales. Criterion validity was addressed using the variables asthma, somatisation, and depression, for which associations with chemical intolerance have been reported. These variables were dichotomized using the ECRHS asthma criteria and the gender-based cut-off scores for the SCL-92 somatisation and depression subscales described by Olsen and colleagues [31, 32]. Further, cross-validation was performed by randomly dividing the data set in two and comparing results with those obtained for the entire data set.

### 3.2. Differential Item Functioning

Differential item functioning (DIF) is the phenomenon that performance of items differs across subpopulations or that items measure different things for members of one subpopulation as opposed to members of another. Instruments containing such items may have reduced validity for between-group comparisons because scores may be indicative of attributes other than those the instrument is intended to measure [37]. We tested DIF by testing conditional independence given the total score. We used the partial gamma coefficient [38], suggested by Kreiner when items are polytomous [39]. DIF with respect to asthma was tested using the ECRHS criteria, and depressive and somatising individuals were identified using the SCL-92 cut-off scores [31, 32].

Data were analysed using SPSS, version 15.0 for Windows and SAS version 9.2.

## 4. Approval

The study was approved by the Danish Data Protection Agency. According to Danish legislation questionnaire studies do not need approval from an ethics committee.

## 5. Results

### 5.1. Sample Characteristics

Characteristics of the patient- and population samples are shown in Table 1. Due to skewed distributions, the medians for the five QEESI scales (the Symptom Severity, Chemical (inhalant) Intolerances, Other Intolerances, Life Impact Scales, and the Masking Index) are presented. Table 1 also includes sex and age distributions for the two samples, mean and median scores on the two SCL-92 subscales, as well as occupational social class (SES). Table 1 shows that scores on the QEESI and SCL-92 differed significantly in the expected direction between the two samples. In analyses stratified by gender QEESI scores also differed significantly (*P* < 0.0001) between the two groups (data not shown). In terms of QEESI, scores also differed significantly (*P* < 0.0001) between women in the population and patient samples as well as between men (data not shown). In regards to age, the patient sample was significantly older than the population and differences were also seen in relation to SES classifications, which may be a consequence of the differences seen in age.

### 5.2. Reliability

Cronbach alpha coefficients and median scores on the four QEESI scales (Chemical (inhalant) Intolerances, Symptom Severity, Other Intolerances, and Life Impact) are shown in Table 2. The Cronbach alpha coefficients were overall high in both groups (range 0.64–0.94) for all four scales suggesting good internal consistency (Table 2).

Pearson correlation analyses of test-retest reliability showed statistically significant coefficients for the five scales: the Chemical (inhalant) Intolerances Scale (0.94, *n* = 230), the Symptom Severity Scale (0.89, *n* = 234), the Other Intolerances Scale (0.89, *n* = 233), the Life Impact Scale (0.96, *n* = 232), and the Masking Index (0.84, *n* = 234).

### 5.3. Validity

The discriminatory validity of the five QEESI scales is shown in Table 3. In the simple logistic regression analyses, the discriminatory power was the largest for the Chemical (inhalant) Intolerance and the Life Impact Scale, and these two scales were therefore selected for subsequent multivariate analyses. Calculating other pairwise comparisons resulted in lower values than the one specified for the Chemical (inhalant) Intolerance and the Life Impact Scales (data not shown). Including more scales in the analysis did not substantially change the result since the maximum value obtained for all five scales was 0.98 (data not shown).

To test whether other variables found to be associated with chemical intolerance, that is, asthma, somatisation, and depression, would influence the discriminatory validity of the Chemical (inhalant) Intolerance and the Life Impact Scales, other subsequent statistical analyses were performed. Area under the ROC curve was calculated using the ECRHS asthma criteria and the gender-based cut-off scores for the SCL-92 somatisation and depression subscales, as described by Olsen and colleagues [31, 32], in the analyses. The following results were obtained: for the Chemical (inhalant) Intolerance Scale the area under the ROC curve for asthma was 0.93 (95% CI 0.90–0.95), for somatisation 0.89 (95% CI 0.86–0.94) (women) and 0.91 (95% CI 0.88–0.94) (men), and for depression 0.94 (95% CI 0.91–0.97) (women) and 0.94 (95% CI 0.91–0.96) (men); for the Life Impact Scale the area under the ROC curve for asthma was 0.94 (95% CI 0.91–0.96), for somatisation 0.88 (95% CI 0.84–0.93) (women) and 0.91 (95% CI 0.88–0.94) (men), and for depression 0.92 (95% CI 0.88–0.95) (women) and 0.93 (95% CI 0.89–0.95) (men) (data not shown). These results suggest that the area under the ROC curve is slightly lowered with coexisting asthma, depression, or somatisation; nevertheless the discriminatory validity of QEESI is still good. Randomly dividing the data set in two yielded results that corresponded with the results obtained for the entire data set: Chemical (inhalant) Intolerance Scale (OR 1.07, 95% CI 1.05–1.09) and the Life Impact Scale (OR 1.05, 95% CI 1.03–1.07) (Area under the ROC curve 0.98).

Construct validity was tested by analysing differential item functioning (DIF), which investigates if item scores are affected by external variables. DIF was tested in asthmatics and in depressives and somatisers using the SCL-92 cut-off scores for caseness [31, 32]. Only statistically significant results are presented in Table 4.

### 5.4. Sensitivity and Specificity

**Figure 1:**
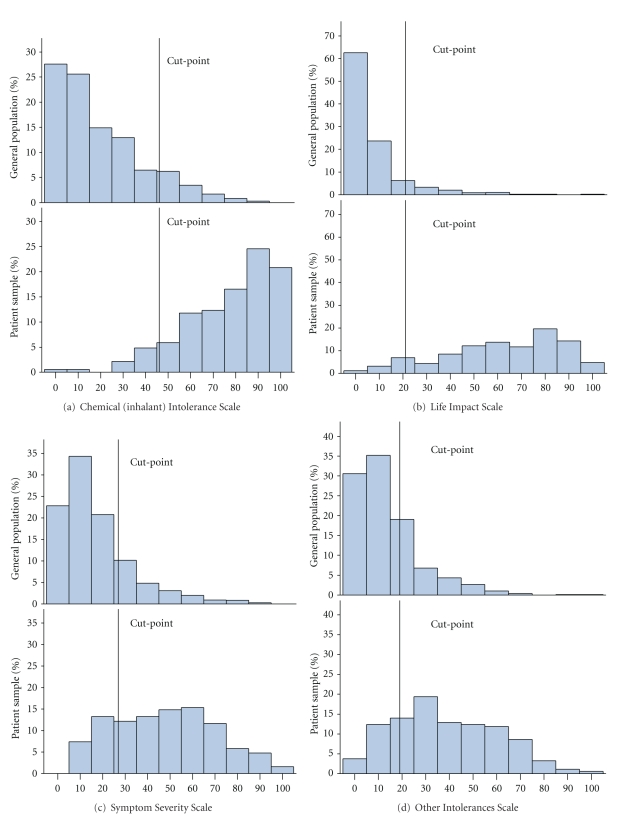
Distribution of the two study samples responses to the four QEESI scales.

**Figure 2:**
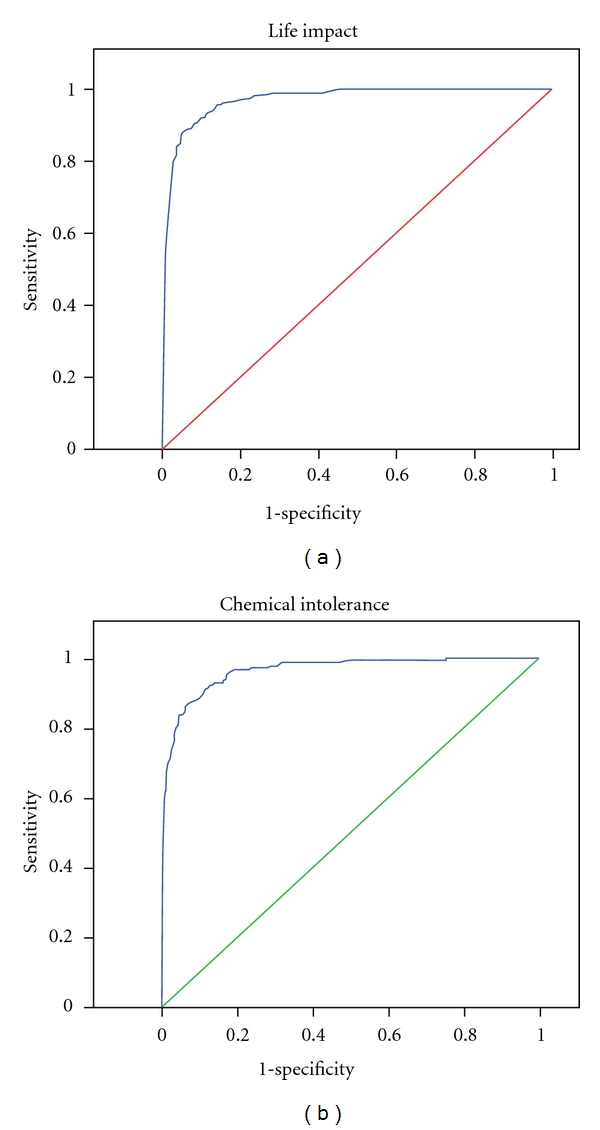
ROC curves for the Chemical (inhalant) Intolerance and Life Impact Scales.

Figure 1 shows the distribution of scores in the two groups and cut-off values for all four scales. Using all scales provided a sensitivity of 92.1% and a specificity of 93.1%. The ROC curves for the Chemical (inhalant) Intolerance and Life Impact Scales are shown in Figure 2. The 95% sensitivity and specificity and optimal cut-off scores for the Chemical (inhalant) Intolerance and the Life Impact Scales when used separately or when combined are shown in Table 5. When used separately, the cut-off values that provided the highest sensitivity and specificity for the two scales were 47 and 21 respectively (Table 5). Combining the two scale scores by using cut-offs of 35 (Chemical Intolerance scale) and 14 (Life Impact Scale) provided highest sensitivity and a specificity (Table 5). Miller and Prihoda [25, 26] used logistic regression to estimate a weighted sum of QEESI scales and an interaction term (the product of two scales) that could be used to provide an optimal cut-point. We used logistic regression finding no significant interactions. In our data this weighted approach yields a sensitivity of 94% and specificity of 91% by first computing *R* = −3.9665 + 0.0619*  chemicalintolerance − 0.0342* otherintolerance + 0.6104* maskingindex + 0.0767* lifeimpactscale − 0.00242* symptoms and then computing the predicted probability prpr = exp⁡(*R*)/(1 + exp⁡(*R*))  and classifying a subject as “chemically sensitive” if prpr > 0.09. These analyses suggest that the difference between our approach and Miller and Prihoda's is minimal. 

## 6. Discussion

The evaluation of the Danish version of QEESI suggested good reliability for the four scales, that is, Chemical (inhalant) Intolerances, Life Impact, Symptom Severity, and Other Intolerances, in terms of internal consistency and coefficients between test and retest scores. 

The overall response rate to the first questionnaire was 64.5%. For the sample characteristics, the patients were significantly older than the general population sample and differences were also found in relation to SES, which may be a consequence of the age differences. In accordance with results reported in other studies, the patient group also scored significantly higher on the SCL-92 subscales [18, 40]. Scores on the QEESI differed between the samples in the expected direction as the patients scored significantly higher on all four scales, that is, the Chemical (Inhalant) Intolerances, Life Impact, Symptom Severity and Other Intolerances Scales, whereas the population scored significantly higher on the Masking Index. 

Our results on the Cronbach alpha coefficients for all four scales and test-retest reliability showed good internal consistency and reliability and correspond to the results obtained in other studies evaluating the QEESI. The Cronbach alphas obtained in this study ranged from 0.64 to 0.94 in the population sample with a tendency to lower scores in the youngest age group, whereas the range in the patient sample was 0.83 to 0.91. Miller and Prihoda reported a corresponding range of 0.89–0.97 for the original American version of the questionnaire [25, 26]. Evaluating a Swedish version of the QEESI, Nordin and Andersson reported a range of 0.74 to 0.95 [27], and in the Japanese version the range was 0.87 to 0.94 for the Chemical (inhalant) Intolerances, the Life Impact, and the Symptom Severity scales [28]. 

The discriminatory validity was the largest for the Chemical (inhalant) Intolerance and Life Impact Scales. Testing the influence of other variables, that is, asthma, depression, and somatisation, by calculating area under the ROC curve did not substantially change the results. Using combined cut-off scores of 35 for the Chemical (inhalant) Intolerance Scale and 14 for the Life Impact Scale provided the best simultaneous sensitivity and specificity, that is, 92.1 and 91.8, respectively. The corresponding sensitivity and specificity for all five QEESI scales were 92.1% and 93.1%. Miller and Prihoda found that the discriminatory power for the Symptom Severity and the Chemical (inhalant) Intolerance Scales was largest [26]. They reported a sensitivity of 83.2% and a specificity of 84.2% using a cut-off score of ≥40 for the Chemical (inhalant) Intolerance Scale [26]. Using the cut-off scores collectively for the Chemical (inhalant) Intolerance Scale (≥40), the Symptom Severity Scale (≥40) and the Other Intolerance Scale (≥25) provided a sensitivity of 67.2% and a specificity of 90.9% [26]. In the Japanese evaluation of three of the QEESI scales, Hojo et al. reported the highest discriminatory ability for the Symptom Severity Scale with a cut-off score of ≥20, which provided a sensitivity of 84.8% and a specificity of 84.0% [29]. Sensitivity and specificity for the Life Impact Scale were 84.8% and 85.7%, respectively, with a cut-off score of ≥10. Contrary to our findings and the findings by Miller and Prihoda, the Japanese version of the Chemical (inhalant) Intolerance Scale had a low sensitivity (73.4%) and specificity (69.6) using a cut-off score of ≥40 [29]. The cut-off scores applied in the Japanese study were defined uniquely for the Japanese translation. Nordin and Andersson reported good discriminatory power for the Symptom Severity, Chemical (inhalant) Intolerance, and Life Impact Scales [27]. The different findings may reflect cross-cultural differences in the responses to QEESI or, perhaps more likely, reflect differences in study populations in relation to the selection and definition of cases. Nevertheless when applying the QEESI in epidemiological studies or in clinical research, our results suggest that using the combined cut-offs scores for the Chemical (inhalant) Intolerance and the Life Impact Scales provides a shorter and equally strong alternative. 

Construct validity was tested by analysing differential item function (DIF). Item function is supposed to be invariant of other, and in this regard, irrelevant constructs [41]. Our analyses suggested that scores on several items on all five scales were influenced if the respondent had asthma according to the ECRHS criteria or had scores above the cut-off values for caseness on the SCL-92 subscales, which may have a negative impact on the construct validity of the Danish translation of QEESI. The use of the ECRHS definition on asthma has been validated with bronchial hyperresponsiveness to methacholine (BHR) [33] but not validated among individuals with chemical intolerance and might therefore overestimate a correlation. However, positive correlations between asthma and chemical intolerance have been described in studies using other self-reported asthma definitions [7, 42] as well as objective measurements of BHR [43]. Using the standards for interpretation of DIF applied by Bjorner et al. [41], the magnitude of DIF for the three items on the Chemical (inhalant) Intolerance Scale, that showed indication of DIF, was none or negligible. The same applied for the Life Impact Scale except for one item (choice of clothing) in relation to depression, which was in the slight to moderate range. However, taken together the magnitude of DIF appears to be of little importance for the construct validity of these two scales. For the remaining three scales the sizes of the gamma coefficients suggested that DIF may be a problem. However, this study is the first to test DIF in relation to the QEESI. Therefore, we cannot compare our results with others and thereby determine whether DIF occurs in other translations of the questionnaire than the Danish version. Accordingly, we recommend that future studies on QEESI address this issue. Altogether our results provide additional evidence of the reliability and validity of QEESI as a clinical survey tool for MCS. The size of the study and the response rate to the questionnaire on both the first and the second test occasion support the validity of our results. However, like most questionnaire-based studies, the information gathered relies upon self-reported and retrospective data, which must be kept in mind when interpreting the results. While reliability and validity of the different translations of QEESI have proven to be good and thereby support the use of the questionnaire in future studies, differences in the case definitions applied in the studies still point to difficulties in the comparisons of results across countries. As stated by Miller and Prihoda in their study published in 1999, the lack of a uniform approach for identifying individuals with chemical intolerance is a barrier for progress in this area [26]. Thus more research in this area is needed to establish internationally agreed diagnostic criteria. Meanwhile, the QESSI provides a good research tool with a response format that allows for continuous scores that may also be used in the evaluation of the effectiveness of treatments.

In conclusion, the Danish translation of the QEESI showed overall good reliability and validity, which is in accordance with the results reported in other studies. Our analyses of construct validity suggested that there may be problems with DIF in three of the QEESI scales. As our study is the first to conduct these analyses, we cannot conclude whether this applies only to the Danish translation. We therefore recommend that future studies on QEESI address this issue. For research purposes, we recommend use of the combined Chemical (inhalant) Intolerance and the Life Impact Scales scores, which provided a sensitivity of 92.1 and a specificity of 91.8 in this study.

## Figures and Tables

**Table 1:** Characteristics of the patient and the population samples.

	Population sample			Patient sample			*P-*value^1^
	Men	Women	Total	Men	Women	Total	
	600	705	1305	25	163	188	
Age, mean (sd)	47.4 (14.1)	46.8 (14.3)	47.1 (14.2)	52.4 (14.7)	56.0 (10.8)	55.5 (11.5)	<0.0001

QEESI (median)							*P-*value^2^

Symptoms	9.0	14.0	11.0	35.0	48.0	47.0	<0.0001
Chemical Int.	11.0	15.0	13.0	81.6	82.2	82.1	<0.0001
Other Int.	6.0	12.0	10.0	27.3	35.5	35.0	<0.0001
Life Impact	0.0	3.0	2.0	70.0	64.0	65.0	<0.0001
Masking Index	4.0	4.0	4.0	2.0	2.0	2.0	<0.0001

SCL-92 (median)							

Depression	0.15	0.31	0.23	0.62	0.69	0.69	<0.0001
Somatisation	0.25	0.33	0.33	0.83	1.0	1.0	<0.0001

SCL-92 (mean)							

Depression	0.39	0.53	0.47	1.0	0.86	0.88	—
Somatisation	0.38	0.51	0.45	1.0	1.13	1.12	—

Occupational social class (*n* (%))							*P-*value^3^

							<0.0001
I + II:			224 (17.2)			17 (9.0)	
III + IV:			426 (32.6)			35 (18.5)	
V + VI + VII:			566 (43.4)			124 (65.6)	
Missing:			89 (6.8)			13 (6.9)	

Occupational social class: I + II: professionals and executives and medium-level white-collar employees; III + IV: low-level white-collar employees and skilled workers; V + VI + VII: unskilled and semiskilled workers, individuals receiving pension or disability benefits, and students.

^1^Independent samples  *t*-test for equality of means (total) between population and patient samples.

^2^Mann-Whitney test (total) comparing population and patient samples.

^3^Chi-squared test comparing population and patient samples.

**Table 2:** Median scores and scale reliability coefficients (Cronbach's alpha).

Scale group	*N*	Symptom scale		Chemical intolerance scale		Other intolerance scale		Life Impact scale	
		Median (IQR)**	Alpha***	Median (IQR)	Alpha	Median (IQR)	Alpha	Median (IQR)	Alpha
Patient sample	189	47 (30–64)	0.84	80 (62–91)	0.91	34 (20–53)	0.83	64 (45–80)	0.89
Population	1309	11 (5–23)	0.86	13 (4–30)	0.92	10 (3–19)	0.77	2 (0–8)	0.86

Population*									
−30	201	12 (5–23)	0.83	11 (4–24)	0.87	11 (3–19)	0.64	4 (0–10)	0.74
30–40	218	11 (4–21)	0.85	15 (5–31)	0.93	11 (5–22)	0.79	2 (0–8)	0.86
40–50	288	11 (6–22)	0.89	14 (5–30)	0.92	11 (5–18)	0.79	2 (0–8)	0.90
50–60	312	12 (5–23)	0.86	14 (4–32)	0.94	8 (3–17)	0.78	1 (0–9)	0.87
60−	290	10 (4–23)	0.86	12 (1–29)	0.93	5 (0–17)	0.75	0 (0–5)	0.81

*Population sample grouped by age.

**Interquartile range.

***Cronbach's alpha.

**Table 3:** Discriminatory power of the five QEESI scales either when used alone (univariate analyses) or when combined in a multivariate logistic regression model.

Scale univariate	*P*-value	Odds ratio (95% CI) one-point increase	Area under ROC curve
Symptom severity	<0.0001	1.07 (1.06–1.08)	0.88 (0.85–0.90)
Chemical intolerances	<0.0001	1.11 (1.09–1.12)	0.97 (0.95–0.98)
Other Intolerances	<0.0001	1.07 (1.06–1.08)	0.84 (0.81–0.87)
Life Impact	<0.0001	1.10 (1.09–1.12)	0.97 (0.96–0.98)
Masking Index (rev)*	<0.0001	2.48 (2.16–2.06)	0.81 (0.78–0.84)

Multivariate			
Chemical intolerances	<0.0001	1.06 (1.05–1.08)	0.98
Life Impact	<0.0001	1.06 (1.05–1.07)	

*Scores on the Masking Index were reversed in the statistical analyses.

**Table 4:** Differential item functioning (DIF). Only significant results are shown.

Scale	Item	Asthmatics	Depressives	Somatisers
		*partial γ* * coefficient*		
*Chemical int.*	item 2 (tobacco smoke)	0.17 (se = 0.06)	−0.16 (se = 0.06)	—
	item 4 (gasoline)	−0.23 (se = 0.06)	—	—
	item 8 (tar)	—	0.16 (se = 0.04)	−0.17 (se = 0.06)

*Life Impact*	item 2 (work ability)	—	0.14 (se = 0.06)	—
	item 4 (choice of clothing)	—	−0.31 (se = 0.12)	—
	item 6 (choice of products)	−0.16 (se = 0.08)	—	—
	item 8 (choice of hobbies)	0.20 (se = 0.09)	—	—
	item 9 (relation with spouse)	—	—	−0.19 (se = 0.10)

*Symptom severity*	item 1 (muscle and joint pain)	0.23 (se = 0.06)	−0.19 (se = 0.06)	—
	item 2 (mucosal or respiratory)	0.47 (se = 0.04)	−0.30 (se = 0.07)	—
	item 4 (stomach and digestive)	—	−0.17 (se = 0.07)	—
	item 5 (concentration/memory)	−0.16 (se = 0.06)	0.36 (se = 0.06)	—
	item 6 (tension and nervousness)	−0.15 (se = 0.07)	0.79 (se = 0.04)	—
	item 7 (balance or coordination)	—	0.21 (se = 0.07)	0.24 (se = 0.08)
	item 10 (genital and urinary)	−0.20 (se = 0.06)	—	−0.27 (se = 0.08)

*Other Int.*	item 3 (unusual cravings)	−0.33 (se = 0.06)	—	—
	item 4 (feeling ill after meals)	—	0.42 (se = 0.08)	0.39 (se = 0.07)
	item 6 (feeling ill)	−0.21 (se = 0.08)	0.24 (se = 0.09)	—
	item 7 (alcoholic drinks)	—	0.27 (se = 0.09)	—
	item 10 (allergic reactions)	0.36 (se = 0.06)	−0.21 (se = 0.10)	—

*Masking Index*	item 2 (alcoholic intake)	−0.17 (se = 0.08)	−0.54 (se = 0.09)	—
	item 4 (fragranced products)	−0.51 (se = 0.06)	−0.35 (se = 0.11)	—
	item 10 (routine use of medicine)	0.62 (se = 0.05).	0.72 (se = 0.06)	—

**Table 5:** Sensitivity, specificity, and optimal cut-off values for the chemical intolerance scale and the Life Impact Scale.

Chemical intolerance cut-off scores	Sensitivity (%)	Specificity (%)
37	95.2	82.8
** 47**	**89.3**	**89.4**
58	83.9	95.2

Life Impact Scale cut-off scores	Sensitivity	Specificity

14	95.8	86.2
** 21**	**91.0**	**90.9**
31	86.8	95.2

Combined scale scores	Sensitivity	Specificity

Chemical intolerance cut-off **35**/Life Impact Scale cut-off **14**	**92.1**	**91.8**

## Abbreviation List

MCS: Multiple chemical sensitivity
QEESI: Quick Environmental Exposure and Sensitivity Inventory
SCL-92: Symptom Checklist 92.
